# Prognosis and Treatment After Diagnosis of Recurrent Esophageal Carcinoma Following Esophagectomy with Curative Intent

**DOI:** 10.1245/s10434-015-4840-5

**Published:** 2015-09-03

**Authors:** K. Parry, E. Visser, P. S. N. van Rossum, N. Haj Mohammad, J. P. Ruurda, R. van Hillegersberg

**Affiliations:** Department of Surgery, University Medical Center Utrecht, Utrecht, The Netherlands; Department of Radiotherapy, University Medical Center Utrecht, Utrecht, The Netherlands; Department of Medical Oncology, University Medical Center Utrecht, Utrech, The Netherlands

## Abstract

**Background:**

Strategies for the treatment of recurrence after initial curative esophagectomy are increasingly being recognized. The aim of this study was to identify prognostic factors that affect survival in patients with recurrence and to evaluate treatment strategies.

**Methods:**

A prospective database (2003–2013) was used to collect consecutive patients with esophageal carcinoma treated with initial curative esophagectomy. Locations, symptoms, and treatment of recurrence were registered. Post-recurrence survival was defined as the time between the first recurrence and death or last follow-up.

**Results:**

Of the 335 selected patients, 171 (51 %) developed recurrence. Multivariable analysis identified distant recurrence as opposed to locoregional recurrence [hazard ratio (HR) 2.15, 95 % confidence interval (CI) 1.27–3.65; *p* = 0.005], more than three recurrent locations (HR 2.42, 95 % CI 1.34–4.34; *p* = 0.003), and treatment (HR 0.29, 95 % CI 0.20–0.44; *p* < 0.001) as independent prognostic factors associated with post-recurrence survival. Primary tumor characteristics, including neoadjuvant therapy, histological type, pTN stage, and radicality, did not independently influence post-recurrence survival. Treatment was initiated in 62 patients (37 %) and included chemotherapy, radiotherapy, and/or surgery. Median post-recurrence survival of all patients was 3.0 months (range 0–112). In total, six patients (4 %) were still disease-free following treatment, indicating cure.

**Conclusions:**

In patients treated for esophageal cancer at curative intent, distant recurrence and more than three recurrent locations were independent prognostic factors associated with worse post-recurrence survival, irrespective of primary tumor characteristics. Although survival after recurrence was poor, treatment can prolong survival and can even lead to cure in selected patients.

Esophageal carcinoma is the sixth leading cause of cancer-related mortality worldwide and the incidence is rapidly increasing.^1^,^2^ Multimodality treatment combining neoadjuvant chemo(radio)therapy and surgical resection has improved the prognosis for resectable nonmetastatic disease;^3^ however, more than half of the patients develop recurrence within 3 years after treatment with curative intent.^4^^–^7 The prognosis of recurrent esophageal cancer is poor, with a median survival of 3–10 months after developing a recurrence.^4^,^8^–^10^ Therefore, detecting prognostic factors affecting post-recurrence survival and determining effectiveness of treatment strategies for recurrence are of high importance. Treatment can be attempted in a fair number of patients with recurrent disease and may include chemotherapy, radiotherapy, surgery, or a combination.^9^,^11^,^12^ However, the optimal treatment strategy for esophageal cancer patients with recurrent disease is not yet established and patients respond differently to treatment, with a wide range in long-term survival.^12^

The main aim of this study was to investigate prognostic factors that affect survival in patients diagnosed with recurrent disease after prior esophagectomy with curative intent for esophageal carcinoma. In addition, a second aim was to evaluate the different treatment strategies applied.

## Methods

### Patients

In this single-center cohort study, patients were selected from a prospectively assembled database at the University Medical Center Utrecht, Utrecht, The Netherlands. Between October 2003 and December 2013, a total of 379 consecutive patients underwent esophagectomy with curative intent for esophageal carcinoma. Patients with an unresectable tumor (cT4b) or metastatic disease (M1) detected intraoperatively were excluded (*n* = 22), as were patients deceased within 90 days after surgery or during hospitalization (*n* = 22). Of the remaining 335 patients, 171 were diagnosed with recurrent disease and were included in the current study. All patients were discussed at a multidisciplinary tumor board meeting preoperatively, postoperatively, and after developing recurrent disease. Institutional Review Board approval was obtained, and the informed consent requirement was waived for this study.

### Treatment

Eligible patients with locally advanced disease (cT ≥2 or cN+) and without clinical evidence of metastatic disease (cM0) received either perioperative chemotherapy or neoadjuvant chemoradiation according to the Dutch guidelines. Eligible patients were >18 years of age, had a WHO performance status ≤2, and did not lose >10 % of their body weight. Before 1 June 2012, the standard treatment for patients with esophageal carcinoma consisted of perioperative chemotherapy (epirubicin, cisplatinum, and 5-fluorouracil),^14^ and after that patients underwent neoadjuvant chemoradiation (carboplatin AUC2 and paclitaxel 50 mg/m^2^ weekly during 5 weeks concomitant with 41.4 Gy (23 × 1.8 Gy).^3^ Before 2008, neoadjuvant therapy was not part of the standard protocol and most patients were operated on without this treatment. Patients not eligible for neoadjuvant treatment were treated with esophageal resection alone. After esophagectomy with en bloc lymphadenectomy, all patients underwent gastric tube reconstruction with a left-sided cervical anastomosis.

### Histopathological Analysis

The resected specimens were reviewed by experienced pathologists in accordance with the TNM-7 staging system of the AJCC.^13^ Resection margins were evaluated using the definitions of the College of American Pathologists.^15^,^16^

### Follow-Up and Definition of Recurrence

After esophagectomy, patients were followed at the outpatient clinic with an interval of 3 months in the first year, 6 months in the second year, and 12 months thereafter until discharge after 5 years of follow-up, which consisted of medical history and physical examination. In case of clinical suspicion of tumor recurrence, diagnostic imaging was performed. Recurrence was confirmed by histopathological biopsy or clinical follow-up, and only the initial number and sites of recurrences were evaluated. The pattern of recurrence was classified as locoregional, distant, or a combination of both. Recurrences at the anastomotic site or within the area of previous resection and nodal clearance in the mediastinum or upper abdomen were classified as locoregional recurrence, while distant recurrence was defined as recurrence in distant organs, pleura or peritoneal cavity, or distant lymph nodes. Disease-free survival was defined as the time between the day of surgery and day of recurrent disease, and post-recurrence survival was defined as the time between the first recurrence and death or last follow-up.

### Treatment of Recurrence

Treatment for recurrent disease was discussed at a multidisciplinary tumor board meeting and was recommended if the patient was eligible. General considerations regarding eligibility included patient condition, location of recurrences, prior toxicity from chemotherapy or radiotherapy, and patient’s wish. Treatment consisted of chemotherapy, radiotherapy, and/or surgery focused on tumor reduction. Radiotherapy focused on tumor reduction was defined as radiotherapy with a radiation dose >30 Gy, excluding palliative radiotherapy for bone metastases. In all other cases, patients were treated with best supportive care.

### Statistical Analysis

To assess prognostic factors for post-recurrence survival, univariable and multivariable analyses by means of Cox proportional hazard models were used, providing hazard ratios (HRs) with 95 % confidence intervals (CIs). All variables with a *p* value <0.20 in univariable analysis were entered in a multivariable analysis. Kaplan–Meier survival curves were constructed for the prognostic factors that remained significantly associated with post-recurrence survival in multivariable analysis. A *p* value <0.05 was considered statistically significant. All statistical analyses were performed using IBM SPSS version 21 for Windows (IBM Corporation, Armonk, NY, USA).

## Results

### Patient Characteristics

Median follow-up of the 335 consecutive patients treated with esophagectomy during the study period was 22.0 months (range 2–135). Of all patients, 171 (51 %) developed recurrent disease, and these patients were included in the current study. The clinical and histopathological characteristics of these 171 patients are shown in Table 1. Mean age was 63 years (standard deviation 8.8) and most patients were male (*n* = 131, 77 %). Perioperative chemotherapy was administered in 63 patients (37 %) and neoadjuvant chemoradiation in 35 patients (21 %). The surgical procedure consisted of a transthoracic approach in 132 patients (77 %) and a transhiatal approach in the remaining 39 patients (23 %). Tumor histology was adenocarcinoma in 136 patients (80 %), and histopathology revealed ≥pT3 (*n* = 129, 75 %) and pN+ disease (*n* = 123, 72 %) in the majority of patients. Of all patients who developed a recurrence, 139 (81 %) underwent a microscopically radical (R0) resection.

**Table 1:** Clinical and histopathological characteristics of 171 patients with recurrent disease after esophagectomy with curative intent

	Recurrence (Total = 171) *n* (%)
Gender	
Male	131 (77)
Female	40 (23)
Age, years (mean ± SD)	63 ± 8.8
ASA score	
1	49 (29)
2	95 (56)
≥3	27 (16)
Neoadjuvant therapy	
No neoadjuvant therapy	72 (42)
Chemotherapy	63 (37)
Radiotherapy	1 (1)
Chemoradiation	35 (21)
Surgical approach	
Transthoracic	132 (77)
Transhiatal	39 (23)
Adjuvant therapy	
No adjuvant therapy	137 (80)
Chemotherapy	34 (20)
Histological type	
Adenocarcinoma	136 (80)
Squamous cell carcinoma	34 (20)
Other	1 (<1)
pT stage	
T0	9 (5)
T1	16 (9)
T2	17 (10)
T3	121 (71)
T4a	8 (5)
pN stage	
N0	48 (28)
N1	49 (29)
N2	47 (28)
N3	27 (16)
Number of harvested lymph nodes (median [range])	20 [2–80]
Radicality	
R0	139 (81)
R1	32 (19)

*ASA* American Society of Anesthesiologists, *SD* standard deviation

### Pattern of Recurrence

Median time to recurrence was 9.0 months (range 1–86) and 164 patients (96 %) developed recurrence within 3 years after surgery. The most common presenting symptoms were pain (*n* = 38, 22 %), malaise (*n* = 23, 14 %), dysphagia (*n* = 21, 12 %), and anorexia (*n* = 21, 12 %). The diagnosis of recurrent disease was based on computed tomography (CT) findings in 118 patients (69 %), whereas in other patients the diagnosis was made with either endoscopic ultrasound (EUS), upper endoscopy, positron emission tomography (PET), or magnetic resonance imaging (MRI). The type of recurrence and the number of locations are presented in Table 2. Distant recurrence was the most common type of recurrent disease (*n* = 76, 44 %), and the liver was the most commonly affected site (*n* = 50, 15 %).

**Table 2:** Location and treatment recurrence of 171 patients with recurrent disease after esophagectomy with curative intent

	Recurrence (Total = 171) *n* (%)
Type of recurrence	
Locoregional	27 (16)
Distant	76 (44)
Combined	68 (40)
Location distant recurrence	
Liver	50 (15)
Lung	41 (13)
Abdominal lymph nodes	40 (12)
Retroperitoneal	40 (12)
Bone	30 (9)
Other	123 (38)
Number of locations with recurrence	
1	49 (29)
2–3	62 (36)
>3	60 (35)
Type of management	
Treatment focused on tumor reduction	62 (37)
Chemotherapy	24 (14)
Radiotherapy	11 (6)
Chemoradiation	13 (8)
Surgery	5 (3)
Surgery + chemotherapy	4 (2)
Surgery + radiotherapy	4 (2)
Other	1 (1)
Best supportive care	109 (63)
Condition	63 (37)
Patient wish	29 (17)
Toxicity	4 (2)
Location	4 (2)
Other	6 (4)
Unknown	3 (2)

### Factors Affecting Post-recurrence Survival

**Fig. 1:**
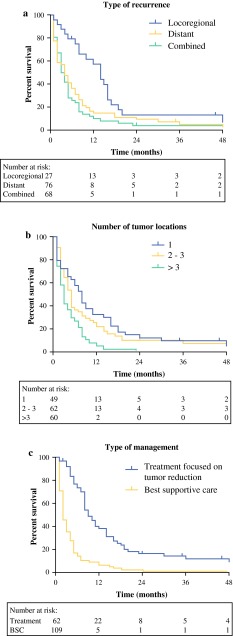
**a** Type of recurrence, **b** number of tumor locations, and **c** type of management were identified as independent prognostic variables for post-recurrence survival in 171 patients with recurrent disease after curative esophagectomy. Survival curves were plotted using the Kaplan–Meier method

Median post-recurrence survival was 3.0 months (range 0–112), and the overall 1- and 2-year post-recurrence survival rates were 17 and 7 %, respectively. Nodal status, type of recurrence, number of locations, time to recurrence, and treatment of recurrence were significantly associated with post-recurrence survival in univariable analysis (Table 3; Fig. 1). In multivariable analysis, distant recurrence (HR 2.15, 95 % CI 1.27–3.65; *p* = 0.005), more than three recurrent tumor locations (HR 2.42, 95 % CI 1.34–4.34; *p* = 0.003), and treatment (HR 0.29, 95 % CI 0.20–0.44; *p* < 0.001) were identified as independent prognostic factors associated with post-recurrence survival (Table 3). The median post-recurrence survival of patients with distant and locoregional recurrence was 2.0 months and 12.0 months respectively. This was respectively 2.0 and 6.0 months for patients with more than three recurrent tumor locations and a solitary recurrence. Patients who received treatment focused on tumor reduction had a median post-recurrence survival of 9.0 months compared with 2.0 months in patients treated with best supportive care. Primary tumor characteristics, including neoadjuvant therapy, histological type, pTN stage, and radicality of resection, did not independently influence post-recurrence survival in multivariable analysis.

**Table 3:** Univariable and multivariable analysis of potential prognostic factors for survival after diagnosis of recurrent esophageal carcinoma

	HR	95 % CI	*p*-Value^a^	HR	95 % CI	*p*-Value^b^
Age (years)	1.02	1.00–1.04	0.055	1.00	0.99–1.02	0.670
Neoadjuvant therapy						
None	Reference	–	–	Reference	–	–
Chemotherapy	1.39	0.98–1.99	0.067	1.02	0.70–1.49	0.936
Radiotherapy	3.45	0.47–25.23	0.222	7.85	0.99–62.54	0.052
Chemoradiation	1.26	0.82–1.94	0.297	0.84	0.50–1.41	0.512
Histological type						
Adenocarcinoma	Reference	–	–			
Squamous cell carcinoma	1.24	0.84–1.84	0.272			
Other	1.10	0.15–7.93	0.922			
pT stage						
T0	Reference	–	–	Reference	–	–
T1–2	0.47	0.21–1.06	0.067	0.60	0.25–1.41	0.243
T3–4	0.70	0.34–1.45	0.341	0.78	0.34–1.76	0.545
pN stage						
N0	Reference	–	–	Reference	–	–
N1	1.80	1.18–2.75	**0.007**	1.50	0.95–2.37	0.080
N2–3	1.35	0.91–1.99	0.131	1.10	0.70–1.73	0.689
Radicality						
R0	Reference	–	–			
R1	1.20	0.81–1.77	0.363			
Type of recurrence						
Locoregional	Reference	–	–	Reference	–	–
Distant	2.10	1.30–3.41	**0.003**	2.15	1.27–3.65	**0.005**
Combined	2.54	1.55–4.16	**<0.001**	1.58	0.89–2.81	0.120
Number of locations						
1	Reference	–	–	Reference	–	–
2–3	1.21	0.81–1.79	0.357	1.30	0.83–2.00	0.250
>3	2.20	1.46–3.32	**<0.001**	2.42	1.34–4.34	**0.003**
Time to recurrence (months)	0.98	0.96–1.00	**0.013**	0.99	0.98–1.01	0.263
Treatment of recurrence						
Best supportive care	Reference	–	–	Reference	–	–
Treatment focused on tumor reduction	0.27	0.19–0.38	**<0.001**	0.29	0.20–0.44	<**0.001**

Analysis was performed using a Cox regression model

Bold values indicate statistically significant (e.g. *p* < 0.05). All variables with a *p* value <0.2 from univariable analysis were used for multivariable analysis

*HR* hazard ratio, *CI* confidence interval

^a^Univariable analysis

^b^Multivariable analysis

### Treatment of Recurrence

Patients receiving best supportive care (*n* = 109, 63 %) were mainly either not eligible for treatment due to a poor performance status (*n* = 63, 37 %) or refused treatment (*n* = 29, 17 %). Some patients were not eligible due to prior toxicity of the neoadjuvant treatment regimen (*n* = 4, 4 %) or tumor location (*n* = 4, 4 %). Treatment focused on tumor reduction was applied in 62 patients (37 %) (Table 2). Patients with locoregional recurrence (*n* = 19, 70 %) and solitary recurrence (*n* = 24, 49 %) more often received treatment focused on reduction compared with those with distant recurrence (26, 34 %) and more than three recurrent tumor locations (*n* = 14, 23 %). Different chemotherapy regimens were administered in 41 patients, with most patients receiving a combination of epirubicin, cisplatin, and capecitabine (*n* = 20, 48 %). After treatment with chemotherapy only, two patients (5 %) showed a clinically complete tumor regression—one patient had a solitary metastasis in the liver, and the other had a solitary locoregional recurrence in the gastric conduit and truncal node. Both patients were alive at last follow-up (35 and 112 months after diagnosis of recurrence).

In 13 of 171 patients (8 %), surgical resection of the recurrence was performed (Table 4), with most of these patients having a solitary recurrence (*n* = 9, 69 %) at a distant location (*n* = 11, 85 %). Surgical resections are outlined in Table 4; five patients (38 %) underwent metastasectomy of a brain lesion. Median post-recurrence survival in patients who underwent resection was 11 months (95 % CI 4.5–17.5), and in 11 of 13 patients (85 %) the resection was performed with curative intent. Of these patients, 4 of 11 (36 %) were still alive at last follow-up, with a follow-up of 5, 46, 53, and 87 months after the diagnosis of their recurrence, whereas the remaining seven patients (64 %) deceased due to disease progression.

**Table 4:** Characteristics, treatment, and survival of 13 patients treated with surgical resection for recurrent esophageal carcinoma

Case	Age, years	Sex	pTNM stage	Time to recurrence (months)	Type of recurrence	Location recurrence	Surgical intervention	Other treatments		Curative intent	Status	Survival after recurrence (months)
								CT	RT			
1	56	Male	T3N2M0	11	Distant	Abdominal LN	LN resection	No	No	Yes	Dead	53
2	44	Male	T3N2M0	3	Distant	Abdominal wall Inguinal cutane	Tumor resection Tumor resection	Yes	No	Yes	Dead	9
3	74	Female	T4aN2M0	2	Distant	Upper leg subcutane Inguinal LN Abdominal wall Abdominal LN	Tumor resection LN resection	No	Yes	No	Dead	4
4	67	Male	T3N2M0	8	Distant	Brain, lung, liver	Metastasectomy brain lesion	No	Yes	No	Dead	5
5	53	Male	T0N0M0	21	Distant	Brain	Metastasectomy brain lesion	No	Yes	Yes	Dead	7
6	77	Female	T3N0M0	14	Distant	Brain	Metastasectomy brain lesion	No	No	Yes	Dead	1
7	75	Male	T1bN0M0	31	Distant	Lung	Partial pulmonary resection	No	No	Yes	Dead	18
8	62	Female	T3N0M0	12	Distant	Brain	Metastasectomy brain lesion	No	No	Yes	Dead	4
9	50	Male	T3N0M0	32	Distant	Vesiculae seminales	Excision vesiculae seminales	No	No	Yes	Dead	11
10	65	Male	T3N3M0	8	Combined	Quadriceps muscles Paraesophageal LN	Metastasectomy quadriceps muscles	Yes	No	Yes	Alive	87
11	56	Male	T2N0M0	13	Locoregional	Gastric conduit	Resection gastric conduit with jejunal reconstruction	No	No	Yes	Alive	46
12	65	Male	T1aN0M0	10	Distant	Liver	Hemihepatectomy	Yes	No	Yes	Alive	53
13	62	Male	T3N0M0	20	Distant	Brain	Metastasectomy brain lesion	No	Yes	Yes	Alive	5

*CT* chemotherapy, *RT* radiotherapy, *LN* lymph node

## Discussion

In this single-center cohort study, 171 patients with recurrent disease after treatment with curative intent for esophageal carcinoma were analyzed and factors affecting post-recurrence survival were evaluated. Distant recurrence and more than three recurrent locations were identified as independent prognostic factors associated with a worse post-recurrence survival, irrespective of primary tumor characteristics. Furthermore, treatment focused on tumor reduction, as opposed to best supportive care, prolonged survival in eligible patients and a selected group of patients were treated curatively.

This study confirms the poor prognosis of recurrent esophageal cancer reported in other series^4^,^8^,^9^,^10^ with a median post-recurrence survival of 3.0 months and a 2-year survival rate of only 7 %. Hence, understanding of the prognostic factors influencing survival is important in identifying patients who could have an improved post-recurrence survival by selecting them for the appropriate treatment. In accordance with the literature, distant recurrence was associated with a worse survival in this study, reflecting aggressive tumor biology.^6^,^12^,^17^ Furthermore, this study showed that patients with more than three recurrent tumor locations had a worse post-recurrence survival compared with those with less involved locations, which could also be explained by the more aggressive behavior of multiple recurrences. The survival of patients with more than three recurrent locations was extremely poor, with a median survival of 2.0 months after the diagnosis of recurrence compared with 6.0 months in patients with a solitary recurrence. The majority of patients had a poor clinical condition at the time of diagnosis of recurrence and were therefore considered ineligible for treatment focused on tumor reduction. The patients who underwent treatment had a significantly prolonged survival, which is likely explained by a combination of appropriate patient selection and treatment effectiveness.

As has been reported in previous studies,^4^,^9^,^18^ all different treatment strategies resulted in a prolonged survival in the current study. This finding suggests that all patients with recurrent disease should be stimulated to undergo treatment if the condition of patients allows it. Median post-recurrence survival in the treated group was 9.0 months compared with 2.0 months for those who were treated with best supportive care. It needs to be acknowledged that the majority of patients who received best supportive care were not eligible for therapy, causing bias through selection-by-indication in this comparison. Nonetheless, most patients who were not eligible had advanced disease (i.e. distant recurrence or more than three recurrent locations), which reflects high dependency of the patient’s condition on the site and number of recurrent tumors.

Patients were treated with various therapies, of which chemotherapy was the most commonly applied. The benefit of a surgical resection of recurrent esophageal carcinoma is not yet completely elucidated. A few reports showed improved survival after surgical resection; 11,19,20 however, in most studies the resection was combined with either chemotherapy or radiotherapy and was performed in only a small number of patients. Also in this study, a small group of patients (*n* = 13) underwent resection of their recurrence, the majority (*n* = 9) of whom had an oligometastasis. Patients with oligometastases represent a special tumor behavior that is likely to gain from local control. In other types of cancer, the current literature also shows a survival benefit with long disease-free survival from local control with surgery for patients with oligometastases.^21^,^22^ Importantly, four patients had complete tumor remission after the resection and were still alive at last follow-up. Other studies also reported long-term survival after treatment of recurrent disease for esophageal carcinoma. 11,23–25 These findings suggest that a favorable outcome can be expected after surgical resection in a selected patient group, especially for those with solitary or localized recurrence of esophageal cancer.

Although treatment of recurrence resulted in prolonged survival, the majority of patients (63 %) received best supportive care. This is in contrast with some other studies where the proportion of patients receiving best supportive care ranged from 12 to 44 %.^9^,^11^,^17^,^26^^,^^27^An explanation for the high percentage of best supportive care in this cohort could lie in the follow-up strategy; the current follow-up strategy is based on the existing literature showing that routine diagnostic imaging is of no benefit with regard to survival and costs.^28^ Furthermore, the consensus-based guidelines from the National Comprehensive Cancer Network also suggest that diagnostic imaging should only be performed when clinically indicated.^29^ Hence, this follow-up strategy is widely performed in The Netherlands; however, it could have resulted in more advanced recurrent tumor stages at the moment of diagnosis. Since the patient’s condition is largely determined by the number and site of recurrences, patients with multiple metastases are often not eligible for therapy; therefore, the follow-up strategy may need revision according to the findings of the current study. In light of new insights into the concept of oligometastases and the new combined treatment options, we suggest routinely performing a follow-up of patients with PET CT in the first 6–12 months following primary treatment.^30^ Another explanation for the high ‘best supportive care’ rate could be the large proportion of patients (27 %) who refused any form of treatment. In most other studies, only a fraction of patients did not receive treatment based on patient’s choice.^17^,^26^,^27^According to the results of the current study, eligible patients might be encouraged to have treatment focused on tumor reduction to improve their survival. Unfortunately, no information on quality of life, which is of paramount importance in patients being treated with palliative intent, was obtained from patients who were treated for recurrence.

## Conclusions

Survival after developing a recurrence after esophagectomy with curative intent is poor. Distant recurrence and more than three recurrent locations were identified as independent factors associated with a worse survival, irrespective of primary tumor characteristics. Treatment focused on tumor reduction using various strategies contributed to a prolonged survival in all patients. Hence, stronger focus is needed to improve patient selection for treatment in recurrent esophageal carcinoma. Additionally, in a small group of patients (4 %), curative treatment of recurrent esophageal carcinoma appears possible.
